# Ethylene Signaling under Stressful Environments: Analyzing Collaborative Knowledge

**DOI:** 10.3390/plants11172211

**Published:** 2022-08-25

**Authors:** Mehar Fatma, Mohd Asgher, Noushina Iqbal, Faisal Rasheed, Zebus Sehar, Adriano Sofo, Nafees A. Khan

**Affiliations:** 1 Plant Physiology and Biochemistry Laboratory, Department of Botany, Aligarh Muslim University, Aligarh 202002, India; 2 Department of Botany, School of Biosciences and Biotechnology, Baba Ghulam Shah Badshah University, Rajouri 185234, India; 3 Department of Botany, School of Chemical and Life Sciences, Jamia Hamdard, New Delhi 110062, India; 4 Department of European and Mediterranean Cultures: Architecture, Environment, Cultural Heritage (DiCEM), University of Basilicata, Via Lanera 20, 75100 Matera, Italy

**Keywords:** environmental stress, ethylene biosynthesis, ethylene signaling

## Abstract

Ethylene is a gaseous plant growth hormone that regulates various plant developmental processes, ranging from seed germination to senescence. The mechanisms underlying ethylene biosynthesis and signaling involve multistep mechanisms representing different control levels to regulate its production and response. Ethylene is an established phytohormone that displays various signaling processes under environmental stress in plants. Such environmental stresses trigger ethylene biosynthesis/action, which influences the growth and development of plants and opens new windows for future crop improvement. This review summarizes the current understanding of how environmental stress influences plants’ ethylene biosynthesis, signaling, and response. The review focuses on (a) ethylene biosynthesis and signaling in plants, (b) the influence of environmental stress on ethylene biosynthesis, (c) regulation of ethylene signaling for stress acclimation, (d) potential mechanisms underlying the ethylene-mediated stress tolerance in plants, and (e) summarizing ethylene formation under stress and its mechanism of action.

## 1. Introduction

Plants are widely exposed to various environmental stresses responsible for the deterioration or alterations in their morphological, physiological, biochemical, anatomical, and molecular characteristics. It has been reported that about 50% of crop loss occurs due to environmental stresses. As a defense strategy, plants develop mechanisms to mitigate the harmful effects of stress, such as the initiation of signaling of different phytohormones [1]. Among the phytohormones, ethylene acts as a stress hormone. However, besides ethylene, ABA is also considered a stress hormone [2]. We may find that under different stresses, these phytohormones might be involved in stress responses and stress adaptations. Ethylene release is triggered by various environmental stresses, such as metal stress, flood stress, and drought stress [3]. It is a simple gaseous hormone that regulates plant growth and development responses under optimal and stressful environments [4,5,6]. It can easily diffuse to nearby cells, and its production occurs locally at the site of its action. Moreover, it has roles in seed germination, nutrient acquisition, senescence, ripening, physiological and molecular mechanisms under optimal conditions, and stress acclimation [1,7,8,9,10,11,12,13].

Several studies have shown that ethylene biosynthesis is significantly induced during fruit ripening, leaf senescence, or under environmental stresses [3,6]. The enzymes 1-aminocyclopropane-1-carboxylic acid (ACC) synthases (ACSs) and ACC oxidases (ACOs) are considered to be the key biosynthetic enzymes that are responsible for ethylene biosynthesis [3]. Ethylene initiates a cascade of adaptive responses in plants under optimal and stressful states. However, the literature does not provide collective information on the induction mechanism of stress ethylene by the influence of environmental stresses or the sources of ethylene production and its exogenous application for stress acclimatization.

Ethylene regulates various mechanisms and interacts with nutrients and/or phytohormones. It has roles in the control of photosynthesis, sulfur (S) and nitrogen (N) and proline metabolism, glycine betaine (GB) production, and the antioxidant defense system to protect plants from environmental stress conditions [10,14,15,16]. Ethylene is involved in synthesizing secondary metabolites [17] responsible for stress tolerance under stressful conditions. Ethylene regulates the glutathione (GSH) synthesis in *Arabidopsis* under ozone stress [18] and in *Brassica juncea* under salt stress [10]. Indeed, ethylene regulates plants’ metabolism for stress tolerance. Thus, ethylene has been connected to the stress response, and data on environmental stresses’ influences on ethylene biosynthesis and signaling are available. However, the picture still needs clarification on many points. We have tried to fill this gap by describing the current knowledge of the mechanism and regulation of ethylene biosynthesis and updating it with the ethylene signaling pathways in plants and their responses to several environmental stresses. The addition of new knowledge of the ethylene response and the clarification of the mechanisms of action will show how plants detect and react to this phytohormone, as well as how the signal is combined with other responses under different environmental stresses, eventually deciding the phenotype of the plant.

## 2. Biochemistry and Molecular Studies on Ethylene Biosynthesis

**Figure 1:**
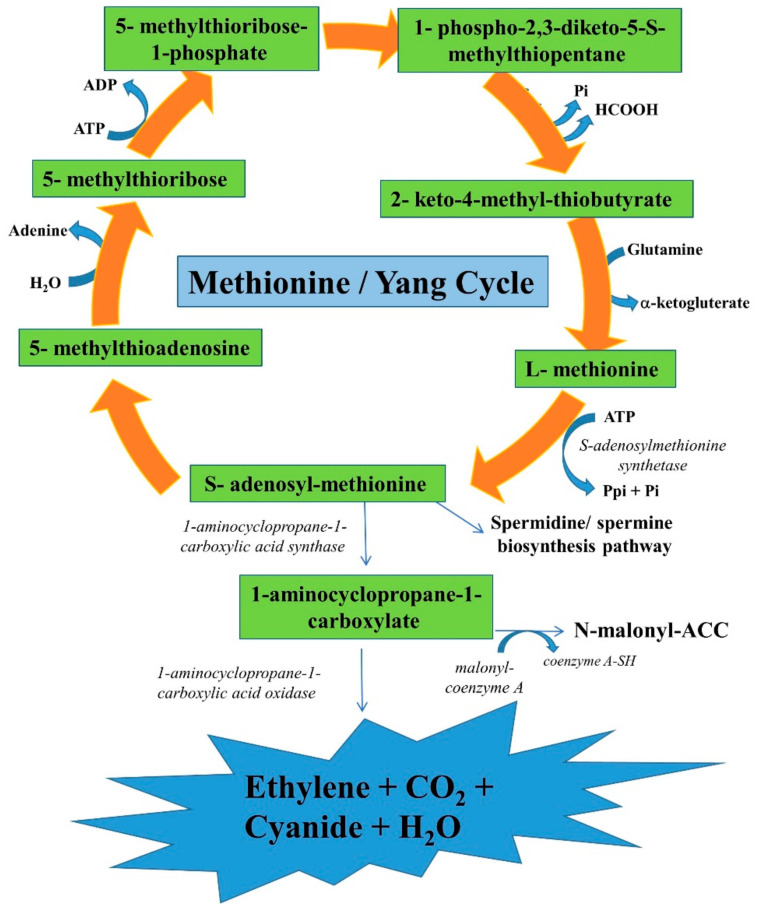
Ethylene biosynthesis with the methionine precursor and the intermediate synthesized as 1-aminocylopropane-1-carboxylic acid (ACC) in higher plants through the Methionine/Yang cycle. Ethylene biosynthesis is the conversion of S-adenosyl-methionine (SAM) from methionine to ACC by ACC synthase (ACS). Methionine is reproduced within the Yang cycle. The Yang cycle is a set of reactions that recycle 5-methylthioadenosine to methionine.

It has been reported that all major classes of photosynthetic organisms, such as cyanobacteria, algae, lichen, early diverging land plants, gymnosperms, and angiosperms, produce ethylene. However, the genetic basis of ethylene biosynthesis varies between taxa [19]. A previous study reported that marine algae, mainly green algae, synthesized acrylic acid in considerable amounts, and the decarboxylation of acrylic acid resulted in ethylene evolution. It has also been reported that the ethylene biosynthesis pathway in green algae is very close to the path described in higher plants [20]. The precursors are the same (L-methionine, S-adenosyl-methionine, SAM, and ACC), but the enzymatic complex transforming ACC to ethylene (ACO) is different, being stimulated by cobalt (Co^2+^). Many reports have shown that the biosynthetic pathway of ethylene in higher plants is well established [5,6,9,21,22]. Under optimal conditions, ethylene biosynthesis occurs through a relatively simple metabolic pathway extensively studied in plants [4]. In general, plants, including algae, produce ethylene from the amino acid methionine in two steps [23]. The production begins with the formation of the intermediate SAM from methionine, mediated by SAM synthetase in an ATP-dependent step [22,23]. In the first step, the substrate SAM is converted to ACC and 5′-methylthioadenosine (MTA) by the enzyme ACS [24,25]. SAM is formed when an adenosyl group is transferred from adenosine triphosphate (ATP) to methionine. Methionine is a sulfur-containing amino acid, and the adenosine binds to the sulfur of the methionine to form SAM with the help of the enzyme SAM synthetase. ACC is produced from SAM by ACS in the next step, and methionine is reproduced within the Yang cycle [26,27] (Figure 1). Besides ACC, ACS also produces 5′-methylthioadenosine in this reaction, which is transformed to methionine through a changed methionine cycle [28], and reserves the methyl group for another round of ethylene synthesis. After that, methionine is converted to decarboxylated SAM, which is catalyzed by the enzyme SAM decarboxylase [29]. The immediate ethylene precursor, ACC, can move basipetal in the phloem or acropetally through the xylem in plants. A recent study demonstrated that the direct ethylene precursor ACC is transported through the xylem via the LYSINE HISTIDINE TRANSPORTER (LHT1) or conjugated into malonyl-ACC or jasmonyl-ACC, which are also transported through the xylem [30]. The last step of ethylene biosynthesis from ACC is catalyzed by ACO and needs O_2_ and carbon dioxide (CO_2_) as a co-substrate and essential activators, respectively [31]. The ACO oxidizes ACC to ethylene and cyanoformic acid, which decarboxylates naturally to cyanide and CO_2_. This poisonous cyanide is detoxified by β-cyanoalanine synthase [32], and ethylene can be degraded by oxidation to ethylene oxide (ethylene monooxygenase) and then to ethylene-glycol [33]. Thus, it can be summarized that the pathway of ethylene synthesis starts from Met to SAM via SAM synthetase, then to ACC via ACS, and ACC to ethylene via ACO. Thus, the two major enzymes for ethylene manipulation are ACS and ACO, and ACC is the immediate ethylene precursor. In higher plants, ACS and ACO enzymes are encoded by multigene families, but these genes are unavailable in the genomes of early diverging plant lineages [34]. Interestingly, several plant lineages deviate from the ACC-dependent pathway for ethylene production. For example, ethylene is synthesized via a distinct metabolism process in aquatic angiosperms belonging to the family “Araceae” [35]. Seed plants of the Ginkgoales and Cycadales use an ACC-independent synthesis route because of the lack of ACO activity [36]. The ethylene biosynthesis trait is present in many fungi and bacteria [37]. The presence of the ACC pathway has been rarely observed outside of plants [38], except in slime molds [39], several lichen mycobionts [40], and the *Penicillium* genus of fungi [38]. Ethylene is produced in all higher plants and is usually associated with a triple response. The triple response of dark-grown pea seedlings to ethylene includes increased stem thickening, reduced stem elongation, and a horizontal growth habit [41,42]. In etiolated pea seedlings, Shaharoona et al. [43] showed that rhizobacterial ACC deaminase, produced in response to various biotic and abiotic stresses, extenuates the ACC-induced classic triple response. Although the ethylene biosynthesis mechanisms under environmental stress are the same as those under optimal conditions, the effect on ACC of different stresses determines the level of ethylene synthesis.

When plants are exposed to environmental stress conditions, an accumulation of the reactive oxygen species (ROS) occurs. This leads to the activation of the mitogen-activated protein kinase (MAPK) cascade in their response and the activation of the ACS and ACO enzymes. The *Arabidopsis* genome encodes nine *ACS* genes, among which *ACS2*, *ACS4-9*, and *ACS11* encode functional ACS, while *ACS1* encodes catalytically inactive enzymes or nonfunctional homodimers [44]. The presence of eight functionally active ACS enzymes in *Arabidopsis* and their ability to form active heterodimers might increase the versatility of ethylene responses, enhancing the capacity to regulate ethylene production after different developmental and environmental stages [45,46]. Non-functional heterodimerization plays a regulatory role in the plant life cycle [46]. The regulation of ethylene biosynthesis has been reported for both positive and negative feedback in several plants [23,28,47]. A study on tomatoes showed that *LE-ACS2* and *LE-ACS4* were positively regulated, and *LE-ACS6* was negatively regulated by ethylene synthesized during fruit ripening [48]. Indeed, the expression of several *ACS* genes is upregulated by various environmental stresses that induce ethylene synthesis [49]. Joo et al. [50] found that ACS2 and ACS6 (isoforms of ACS in *Arabidopsis*) act as substrates for the MAPK cascade, and the phosphorylation of ACS2/ACS6 by MPK6 leads to ACS protein accumulation, which results in ethylene induction. In contrast, unphosphorylated ACS6 protein is degraded by the 26S proteasome pathway, which decreases ethylene production. This suggests the post-translational modification for ethylene formation under stress. The mutants *eto1*, *eto2*, and *eto3* have a constitutive ethylene response phenotype due to ethylene overproduction. These dominant mutations cause increased stability in their corresponding ACS proteins [51], suggesting that ACS enzymes are the target of protein degradation [52,53]. It was also reported that the phosphorylation of MAPK sites in type 1 ACSs increased protein stability and, as a result, increased ethylene production [50,51]. Indeed, several studies suggested that an essential component of ACS and ethylene biosynthesis regulation occurs post-transcriptionally by stabilizing ACS proteins [54,55,56]. Interestingly, the stability of type 2 and type 3 ACS proteins is dependent upon RING-type E3 ligases’ activity [56]. The study by Wang et al. [55] reported that *Arabidopsis* root produces ethylene due to the loss of function of ETHYLENE OVERPRODUCER 1 (ETO1). They reported that the *ETO1* mutation (*eto1*) is in a gene that negatively influences ACS activity and ethylene production. The ETO1 protein interacts directly with and inhibits the enzyme activity of full-length *ACS5* and results in ACS5 protein and ethylene accumulation. *ETO1* overexpression inhibits the induction of ethylene production by cytokinin (plant growth regulator) and promotes *ACS5* degradation by a proteasome-dependent pathway [55]. The *ETO1* gene encodes a ubiquitin E3 ligase that controls ethylene biosynthesis by modulating the levels of *ACS5* [57], and *ETO1* serves as a substrate-specific adapter protein [55]. Here, ETO1 performs a dual mechanism, inhibiting the activity of the ACS enzyme and targeting protein degradation, and permitting the rapid modulation of the enzyme concentration. Moreover, *Arabidopsis* also contains two *ETO1* paralogs, *ETO1-LIKE1* (*EOL1*) and *ETO1-LIKE2* (*EOL2*). Likewise, ETO1, EOL1, and EOL2 contribute to the ubiquitin-dependent degradation of the type 2 ACS proteins or interact with ACS5 to decrease its activity [55,56]. Reports by Chae et al. [58] and Hansen et al. [54] suggested that the *eto2* and *eto3* mutations only partially disrupt the binding of ACS5 and ACS9 to ETO1/EOL1, and that hormones, cytokinin, and brassinosteroid further prevent this interaction. ETO1 and EOL1 do not interact with the *Arabidopsis* type 3 ACS (ACS7) [59]. Another XBAT32 is a RING-type monomeric E3 ligase that regulates ethylene homeostasis. XBAT32 was recently discovered to be necessary for the degradation of ACS4 and ACS7. It has been reported that *1* mutants overproduce ethylene and exhibit various ethylene-related symptoms, including a mild triple response in dark-grown seedlings, reduced plant height, and a reduction in the number of lateral roots [56]. Similar to ETO1, EOL1, and EOL2, XBAT32 is involved in the ubiquitin-dependent turnover of ACS4, but E3 does not interact with ACS11, so XBAT32’s role in regulating the abundance of type 2 ACS protein may be limited [60]. Thus, the ACS enzyme is regulated by distinct transcriptional and post-transcriptional inputs and allows full control of ethylene biosynthesis in plants under various developmental and environmental states.

However, it is equally relevant to determine the importance of ACO in ethylene synthesis. ACO activity is increased under salinity stress, which leads to increased ethylene in *Cicer arietinum* root [61]. The transcripts of *ACO1* in wheat were found to decrease under salinity and other abiotic stresses, resulting in lower ethylene formation [62]. The constitutive expression of wheat *ACO1* in *Arabidopsis* led to increased expression of *AtMYB15* and suppression of the *AtRAB18*, *AtCBF1*, and *AtCBF3* genes, which are stress-responsive and, therefore, salt-sensitive [63].

We might be baffled by ethylene’s different responses under different stresses. However, it is equally relevant to know the cause of the ethylene response. To summarize the knowledge, we may say that ethylene under different stresses may be a positive or negative regulator of stress tolerance. Still, its presence under stress is unequivocal—it activates differential responses in plants. Where it is involved in tolerance, it triggers the induction of antioxidative enzymes and stress-related gene expression, and may work in coordination with different hormones for alleviating stress. In case of sensitivity to stress, excess ethylene up-regulates ROS production and senescence, and growth reduction. However, even in these cases, the reduction effect on plant growth could be a survival strategy for adaptation. An increase in ROS has now been proved to act as a signal under different stresses to alarm the plant of the stress it will encounter and prepare the defensive machinery accordingly. Regardless of whether the stress is low or high, ethylene production occurs in both cases. Even under non-stress conditions, ethylene formation occurs through the formation of ACC from ACS, and then its conversion to ethylene via ACO.

**Figure 2:**
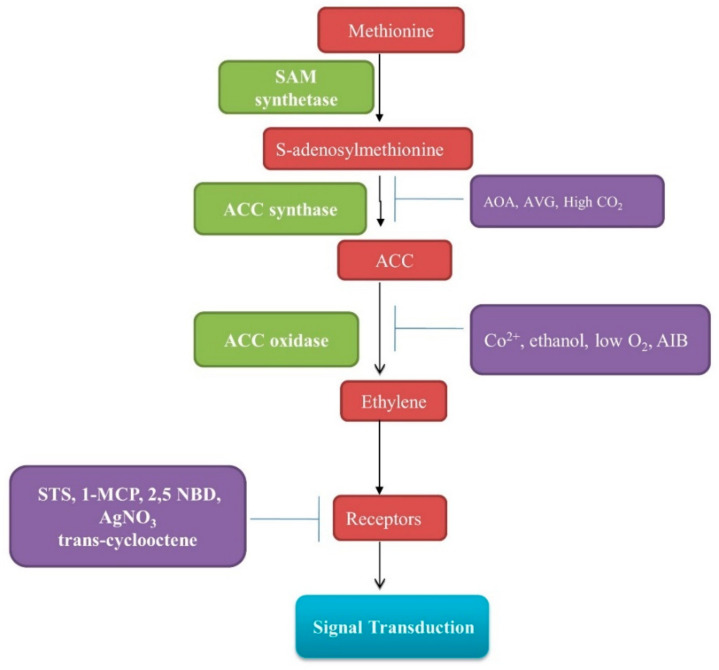
Ethylene biosynthesis in plants and its biosynthetic and action inhibitors. *Biosynthetic inhibitors*: AOA, aminooxyacetic acid; AVG, aminoethoxyvinylglycine; AOIB, 2-aminooxyisobutryic acid; Co^2+^, cobalt; ethanol; and low O_2_ *Action inhibitors*: Silver nitrate (AgNO_3_) or silver thiosulfate (STS), 1-methylcyclopropene (1-MCP), norbornadiene (NBD), and trans-cyclooctene.

Ethylene biosynthesis and its action are inhibited to improve the shelf life of products. The commonly used ethylene biosynthesis inhibitors are 2-aminoethoxyvinyl glycine (AVG) and aminooxyacetic acid (AOA), and ethylene action inhibitors include 1-methylcyclopropene (1-MCP), norbornadiene (NBD), silver thiosulfate (STS), silver nitrate (AgNO_3_), etc. AVG is an inhibitor of ACS, while 1-MCP and silvers are inhibitors of ethylene receptors (Figure 2). Ethylene inhibitors block ethylene biosynthesis or ethylene action; therefore, fruits, plants, and flowers do not respond to endogenously produced ethylene or ethylene from exogenous sources.

## 3. Ethylene Signaling

Ethylene signaling is well known to regulate plant growth and development. This signaling was predominantly delineated with research on *Arabidopsis thaliana*, and the information from *Arabidopsis* about ethylene signaling is usually applied to other plant species [64]. In *Arabidopsis*, ethylene is perceived by the family of receptors ETHYLENE RESPONSE1 (ETR1), ETR2, ETHYLENE RESPONSE SENSOR1 (ERS1), ERS2, and ETHYLENE INSENSITIVE4 (EIN4), which act negatively in ethylene signaling, are related to two-component histidine kinase receptors [65,66], and are located at the Golgi and endoplasmic reticulum (ER) membranes [16,67]. Ethylene is a gaseous hormone that may diffuse in both aqueous and lipid environments [10], so its perception does not require a plasma membrane-localized receptor. Several studies have suggested that ethylene receptors are located on the ER membrane [68,69]. Therefore, once ethylene is biosynthesized, it freely diffuses throughout the plants through plasma membranes, reaching the ER, and binds to the ethylene receptors anchored in the ER membrane to stimulate ethylene responses. In ethylene’s absence, ethylene receptors activate the CONSTITUTIVE TRIPLE RESPONSE1 (CTR1), a Raf-like Ser/Thr protein kinase [70,71]. Interestingly, CTR1 phosphorylates the C-terminal end of EIN2 (EIN2-CEND) and turns off EIN2, an ER-localized membrane protein. The mRNAs for F-box proteins, EBF1 and EBF2, are translated in the cytosol and target the master transcriptional regulators of ethylene signaling, EIN3 and EIN3-LIKE1 (EIL1), to proteasomes for protein turnover in the nucleus. Thus, no ethylene-stimulated transcriptional responses are activated. In the presence of ethylene, ethylene binding to their receptors shuts off, and CTR1 becomes inactivated, EIN2 is dephosphorylated and cleaved, and it releases C-terminus (EIN2-CEND), which enters the cytosol (moves to P-bodies) and nucleus. In the cytosol, EIN2-CEND represses the translation of EIN3 BINDING F-BOX PROTEIN1 (EBF1) and EBF2 transcripts by directly or indirectly binding to their 3′-untranslated regions (3′-UTRs) and moving along with these transcripts to the P-bodies [72,73]. In the nucleus, EIN2-CEND directly or indirectly promotes the activity of EIN3 and EIL1. Ethylene stabilizes the EIN3/EIL1 transcription factors and regulates the transcription of ethylene-responsive target genes, such as ETHYLENE RESPONSE FACTOR (ERF) [22,74,75]. It was reported that a gene from the ERF family (isolated from tomato), *LeERF1*, positively modulates the ethylene triple response on etiolated seedlings, plant development, fruit ripening, and softening, as it was shown that *LeERF1* is used transgenically in tomato of sense *LeERF1* *(LeERF1*-sn), wild type, and transgenic tomato of anti-sense *LeERF1* *(LeERF1*-as) tomato [76].

**Figure 3:**
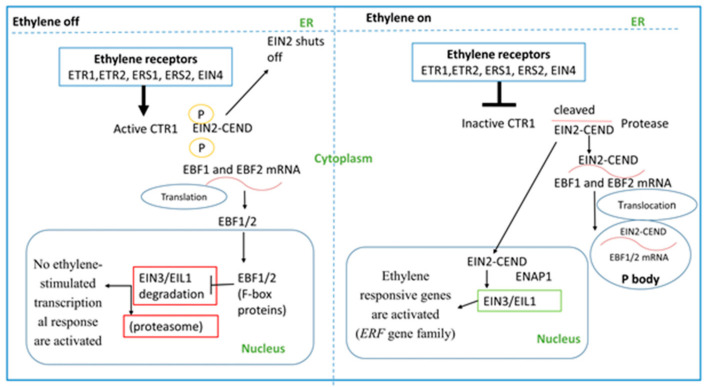
Mechanism of the ethylene signaling pathway in plants. Ethylene receptors ETHYLENE RESPONSE1 (ETR1), ETR2, ETHYLENE RESPONSE SENSOR1 (ERS1), ERS2, and ETHYLENE INSENSITIVE4 (EIN4) are present on the endoplasmic reticulum (ER) membrane. In the absence of ethylene, ethylene receptors activate the CONSTITUTIVE TRIPLE RESPONSE1 (CTR1) protein kinase, which, in turn, phosphorylates the C-terminal end of EIN2 (EIN2-CEND) and turns off EIN2’s function. In the cytosol, the m-RNA F-box proteins EBF1 and EBF2 (EBF1/2) are translated and target the transcription regulation of ethylene signaling. ETHYLENE INSENSITIVE3/ETHYLENE INSENSITIVE3-LIKE 1 (EIN3/EIL1), functioning in the proteasome for protein turnover, is degraded in the nucleus, thus preventing ethylene responses. When ethylene is available, ethylene receptors no longer activate CTR1, dephosphorylate EIN2, and cleave, thus releasing EIN2-CEND, which functions in the cytoplasm and nucleus. In the cytoplasm, one fate of EIN2-CEND is to bind the RNAs for EBF1 and EBF2, become sequestered in processing bodies (P-bodies), and inhibit their translation. The other fate of EIN2-CEND is that it shuttles into the nucleus and directly or indirectly potentiates the activity of EIN3 and EIL1 via EIN2 nuclear-associated protein 1 (ENAP1). In the nucleus, EIN3/EIL1 regulates the transcriptional cascade involving transcriptional factors.

The EIN2-CEND also contributes to the downstream signaling of ethylene via the elevation of the acetylation of histone H3K14 and the non-canonical acetylation of H3K23, as reported by Zhang et al. [77]. EIN2-CEND regulates H3K14Ac and H3K23Ac in response to ethylene and uncovers the unique mechanism by which EIN2 nuclear-associated protein 1 (ENAP1) interacts with histone in the absence of ethylene, preserving the open chromatin status and enabling a rapid response to ethylene stimulation; however, in the presence of ethylene, EIN2 interacts with ENAP1, elevating the levels of H3K14Ac/H3K23Ac, promoting more EIN3 proteins to bind to the target shared with ENAP1 and resulting in rapid transcriptional regulation [78]. Another ethylene receptor, ETR1, is an example of a hybrid histidine kinase, because it contains a histidine kinase domain and a receiver domain; in contrast, ERS1 contains only a histidine kinase domain [73]. CTR1 interacts with both the histidine kinase and receiver domains of ETR1 and the histidine kinase domain of ERS1 [79]. Testerink et al. [80] observed that phosphatidic acid blocked the interaction of CTR1 with ETR1, which is an ethylene receptor, by inhibiting CTR1 kinase activity. The mechanism of the ethylene signaling pathway in plants is shown in detail in Figure 3.

## 4. Influence of Environmental Stress on Ethylene Biosynthesis and Signaling

### 4.1. Induction Mechanism of Stress Ethylene by the Influence of Environmental Stress

Stress ethylene refers to the accelerated biosynthesis of this hormone induced by environmental stresses. Ethylene stress is crucial to plant adaptation and survival under environmental stresses; however, ultimately, it leads to plant death. The underlying mechanism of how these stresses influence ethylene biosynthesis has been reported in various studies, but we are systematically updating the current information on the influence of major environmental stresses, such as heat stress, heavy metal stress, salinity stress, drought stress, and flood stress, on the induction mechanism of stress ethylene and its responses in plants under one roof.

Heat stress may induce or reduce ethylene formation, depending on the activation or suppression of ACS activity. A study reported that exposure to heat stress accelerated the production and accumulation of ethylene in kiwifruit [81], while it decreased those in some plants, such as tomato (*Lycopersicon esculentum*) [82]. Heat stress causes excessive accumulation of ROS, causing oxidative stress. The increase in ROS to a certain level triggers a signal for ethylene synthesis. Oxidative stress, especially due to hydrogen peroxide (H_2_O_2_), acts together with ethylene in a self-amplifying feedback loop where ethylene-induced H_2_O_2_ accumulation enhances ethylene production and H_2_O_2_ initiates leaf senescence and chlorosis under heat stress [83]. Ethylene synthesis in heat stress has been associated with decreased pollen germination and development [84]. Contrarily, an ethylene biosynthesis inhibitor, AVG, leads to the reduction of chlorophyll and induces electrolyte leakage under heat stress in *Lolium perenne* [85]. Moreover, heat stress differentially modifies ethylene biosynthesis and signaling in pea floral and fruit tissues. Under heat stress, the up-regulation of ethylene biosynthesis gene expression in pre-pollinated ovaries is associated with higher ethylene evolution and lower retention of these fruits [86]. Several reports showed that ACS regulation is focused on *ACS* gene expression in response to various environmental stimuli, and its enzymes are spatially and temporally regulated and controlled by several external and internal signals. At high temperatures (about 35 °C), there is no change in the ethylene concentration in creeping bentgrass [87]. Therefore, this suggests that heat tolerance and the heat stress responses for stress ethylene production vary between different plant species, and ethylene exhibits time and dose-dependent effects on the plants during heat stress [88].

A heavy metal-induced increase in ethylene production has been reported in several plant species, such as *Arabidopsis thaliana*, *Triticum aestivum*, *Pisum sativum*, *Glycine max*, *Brassica juncea*, etc. [19,89]. Ethylene acts as a negative regular in plant responses under heavy metal stress because the plant shows a rapid increase in ethylene production with a subsequent reduction in growth [8]. A study demonstrated that Cd exposure significantly increased ethylene emission and its biosynthetic gene *NnACS* expression. Similarly, heavy metals, such as chromium (Cr) and Cd, increased ethylene production in *Brassica juncea*, *Triticum aestivum*, and *Brassica juncea* [5,8,90]. This was because the oxidative stress produced during Cd stress caused the activation of the MPK3 and MPK6 cascade, which regulated ACS2/ACS6 transcription, thereby contributing to stress-induced ethylene production [54,91]. It has also been noted that both *ACS* and *ACO* genes were regulated independently by specific stresses [28]. Moreover, the upregulation of *ACO* genes led to ethylene production under copper (Cu) stress and induced the expression of *ACO1* and *ACO3* genes in *Nicotiana glutinosa* [92].

Salinity increases ethylene biosynthesis in several lettuce cultivars during the germination phase and in pepper, broccoli, and beetroot. At the same time, a decrease occurs in melon, spinach, and tomato [93,94]. Indeed, studies suggested that salt stress enhances ethylene biosynthesis, but this was stress ethylene that had to be brought down to an optimum level favoring plant photosynthesis and growth. It has been reported that ACC negatively affected tomato seedlings’ growth under salinity stress [93]. Interestingly, it has also been reported that the response of ethylene biosynthesis to salt stress is related to plant sensitivity; for example, pepper shoot is the most sensitive to saline treatment, showing the highest fresh weight inhibition and the highest increase in the total ACC concentration [95]. In contrast, beetroot is less affected by salinity and shows no effect on the total ACC concentration in response to saline treatment [93]. Thus, the greater the resistance of the plant to salinity, the lesser the response to this stressor, including at the level of the production of stress ethylene. Saline treatment increases the total ACC concentration in both the roots and shoots in most of the plant species examined, such as *Capsicum annum*, *Lycopersicon esculentum*, *Brassica oleraceae*, *Lactuca sativa*, *Cucumis melo*, *Phaseolus vulgaris*, *Spinacia oleracea*, and *Beta vulgaris*, which is related to plant sensitivity to salinity [94,95].

Drought is an important abiotic stress factor that limits plant growth and development. Under drought stress conditions, an increased level of ethylene or stress ethylene has been reported in rice plant species [96,97]. During the biosynthesis of ethylene induced by water stress, the de novo synthesis of the enzyme ACS is induced and is responsible for the accumulation of ACC and increase in stress ethylene production [98]. Under drought conditions, ACC acts as a transduction molecule for *OsERF109*, which negatively regulates drought tolerance and reduces ethylene formation, acting as a switch during drought tolerance to avoid excessive ethylene production [97]. It has been reported that potassium (K^+^) starvation inhibits the water stress-induced stomatal closure by favoring the synthesis of ethylene, which interacts negatively with ABA in stomatal closure in sunflower plants [99]. The study by Tanaka et al. [100] suggested that wild-type plants treated with ABA show faster stomata closure than ethylene-supplemented *Arabidopsis* plants.

In the context of climate change, flooding has been identified as significant environmental stress for plant growth and crop production worldwide. It has been noted that, during flooding, the limitation of gas diffusion results in O_2_ shortage and accumulation of ethylene in flooded tissues, and results in the formation of ROS [101]. During flooding, the root releases a high amount of ACC, resulting in stress ethylene production in tomato plants [102]. Interestingly, ACC formed in roots is transported to the shoots during flooding, where it is converted to ACC and ethylene, increasing their production in tomato plants [103]. A recent study by Yamauchi et al. [104] suggested that the growth of rice seedlings under sufficient O_2_ and ethylene inhibitor (1-MCP) under stagnant flooding leads to the suppression of aerenchyma formation. Moreover, it has also been reported that the *ACO* genes *ACO8* and *ACO3* are strongly induced in submerged rice shoots, whereas *ACO1* is negatively regulated. The comparison of the tolerant (M202-*Sub1*) and the intolerant (M202) accessions showed that ethylene is significantly higher in the genotypes that are submergence-intolerant compared with the tolerant genotype [105]. This process is regulated transcriptionally by *ACS* gene *ACS2*, which is repressed in the tolerant genotype. Thus, understanding the biosynthesis of ethylene in flood-tolerant plants could be useful for enhancing the tolerance of food crops.

Thus, in general, under abiotic stress, increased production of ethylene occurs due to the generation of ROS that activates the MAPK cascade, which leads to ethylene formation through the activation of the ACS and ACO genes. However, reports of decreased ethylene production under certain stresses have been reported, which makes the role of ethylene rather specific to the plant type, growth condition, timing, and organ under study.

### 4.2. Regulation of Ethylene Signaling for Environmental Stress Acclimation

Ethylene’s presence affects a plant’s tolerance to various environmental stresses [3] through multiple levels of regulation [22]. Ethylene can differentially regulate photosynthesis and growth to mediate plant adaptation to different environments [106]. It regulates ROS metabolism by modulating enzymatic antioxidants [107]. Ethylene-induced ERFs play an important role in redox regulation [108]. According to Jesperson et al. [109], ethylene plays a vital role in thermotolerance and maintains the functional integrity and stability of plant cells in creeping bentgrass. Interestingly, it was also reported that a low ethylene concentration facilitates the activation of defense signaling in plants, and a high concentration inhibits defense signaling in cucumber and wheat [6,110]. Therefore, these findings suggest that appropriate endogenous ethylene levels and low ethylene sensitivity could efficiently ameliorate heat-induced oxidative stress in different plant species. Moreover, heat shock proteins upregulate *ERF1* overexpression in *Arabidopsis*, and upgrade the tolerance to heat in transgenic lines compared with the wild type by increasing the expression of heat tolerance genes [111]. Ethylene-mediated signaling confers thermotolerance in plants and facilitates the regulation of heat shock factors in rice seedlings [112]. Furthermore, ethylene signaling develops heat resistance and maintains chlorophyll content by reducing oxidative stress or involving genes related to ethylene signaling in plants [112]. Xu et al. [113] reported that *ERF021* exhibits a noted 78.7-fold initiation under heat stress, signifying ethylene’s function in the tolerance to heat stress in soybean. Additionally, pollen development under heat stress upregulates multiple ethylene-responsive genes (*ER5*, *ER21*, *LeJERF1*, and *ER24*) involved in ethylene signaling in *Solanum lycopersicum* [114]. *ERF3* also regulates ROS metabolism in tobacco, resulting in lower accumulation of ROS and enhancing tolerance to various abiotic stresses [115]. The ethylene biosynthesis and signaling-related genes *ACO1*, *ACO4*, *EREB*, and *ETR4* are also significantly up-regulated during heat stress in tomatoes [116]. Interestingly, *etr1* and *ein2* mutants under heat stress exhibited a lower survival rate, suggesting the involvement of ethylene in acquiring thermotolerance in *Arabidopsis* [117]. Stress-induced ethylene acts to trigger stress-related effects in plants. Exogenous ethephon causes thermotolerance through its effect on several stress-related proteins responsible for plant cells’ functional integrity and stability [118]. Ethylene signaling reduces oxidative stress and maintains the chlorophyll content and thermotolerance [112]. Contrarily, the inhibition of ethylene by AVG delays the heat stress-induced effect on leaf senescence by upregulating antioxidant enzyme activities in creeping bentgrass [119]. The overexpression of *ERF1* in *Arabidopsis* improves heat stress tolerance by enhancing the expression of heat-tolerance genes [111].

Reports suggested that, among all of the different metals, Cd was thought to be a more phytotoxic inorganic ion, stimulating ethylene production in plants [120]. A strong line of evidence has shown the multiple facets of ethylene in plant responses under heavy metal stresses, depending upon the endogenous ethylene concentration and ethylene sensitivities that differ in the developmental stages of plant species [121,122]. A recent study suggested that ethylene biosynthesis plays an important role in *Nelumbo nucifera*’s response to Cd stress, maintains appropriate ethylene levels and low ethylene sensitivity, and improves Cd tolerance via efficient antioxidant defenses [123]. Several studies also suggested that the exogenous application of ethylene plays a crucial role in adapting to heavy metal stress in rice [124]. Asgher et al. [125] showed that ethylene promotes Cd stress tolerance in mustard. Singh et al. [126] suggested that the ethylene precursor ACC improves plant tolerance under arsenic (As) stress in *Arabidopsis*. The application of ethephon (ethylene source) significantly decreases oxidative stress, regulates antioxidant metabolism by maintaining a higher level of GSH, and alleviates photosynthetic inhibition under nickel (Ni), and zinc (Zn) stress through the optimization of endogenous ethylene or ethylene homeostasis in mustard [121]. These findings reveal ethylene’s complex and biphasic regulatory function, which likely depends on the endogenous ethylene concentrations under heavy metal stresses, which differ between plant species. The ethylene-regulated photosynthetic processes depend on the sensitivity of plants to ethylene. Thus, ethylene sensitivity has been used as a tool for augmenting the photosynthetic potential of plants under heavy metal stress. Moreover, heavy metals, such as Cd and Cu, increase ACS activity in *Solanum tuberosum* [127], and the optimum level of ethylene synthesis aids in the scavenging of ROS [10]. Optimizing endogenous levels in plants has led to the development of transgenic crops with improved heavy metal tolerance [122]. Decreased ethylene production was confirmed by using *acs2-1acs6-1* double knockout, which positively affected leaf biomass and resulted in a delayed induction of ethylene-responsive gene expressions without significant differences in the Cd contents between wild-type and mutant plants; additionally, the expressions of *ERF1* and *ACO2* in the wild-type is significantly higher compared with those of the mutant in *Arabidopsis* [91]. It has also been reported that EIN2 works as a positive regulator in lead (Pb) resistance in *Arabidopsis* [128]. Ethylene acts critically in enhancing ROS accumulation, playing the role of a signaling agent for provoking defense machinery. It has also been reported that ethylene signaling plays a positive or negative role in seed germination and seedling growth under saline stress. Ethylene receptor ETR2 was found to be a positive regulator involved in boosting seed germination under saline stress conditions in *Arabidopsis* [129]. Exogenous ethylene supplementation improves plant growth and development by strengthening the antioxidant system under salt stress [13,14]. Exogenous ethylene optimizes endogenous ethylene and ACS activity, strengthening the antioxidant system and eventually improving salinity tolerance in mustard [10]. A rise in ethylene production was also found in the salt-adapted callus of sunflower, in which ethylene production is related to stress tolerance [130]. In contrast, salinity does not enhance ethylene production in maize [131]. These findings suggest that the increased rate of ethylene production, as a consequence of salt stress, actually causes some of the symptoms of stress or induces acclimation processes [132]. Moreover, ethylene-mediated molecular mechanisms are implicated in the acclimation response to salt stress through the regulation of free proline accumulation and increased ROS scavenging in *Arabidopsis* [133]. The application of ethylene or its precursor ACC improves plant tolerance to high salinity, largely by increasing the expression of ROS scavengers [62,134]. In other words, ethylene signaling factor EIN3/EIL1 provokes ROS gene utterance to prevent ROS buildup and thus increases salt tolerance because the signaling action of ethylene depends upon the concentration of ROS [135]. A study by Lin et al. [135] indicated that OsWAK112, a wall-associated kinase, negatively regulates plant salt stress responses by inhibiting ethylene production. Additionally, the optimal ethylene level for normal plant growth might vary between different stages and in different plant species, such as *Arabidopsis* and *Oryza sativa* [136]. The overexpression of the metal-binding metalloenzyme encoded by *OsARD1* elevates the endogenous ethylene to reduce the sensitivity of rice plants to salt. This suggests that ethylene plays an important role in salt tolerance in plants [137]. Interestingly, ethylene receptors are negative regulators of ethylene signaling, and the inhibition of ethylene receptors has been observed during salinity stress in several plant species. In *Arabidopsis*, osmotic and salt stress reduce the expression of *ETR1* [6]. It has been reported that the loss of the mutant of the ethylene receptor (*etr*) is associated with enhanced tolerance, while the gain of *etr-1* is associated with increased sensitivity to salinity stress in rice [124]. Salt stress enhances the ethylene receptor *NTHK1* mRNA level in tobacco plants [6]. Several ethylene receptor genes, such as *ETR1*, *ETR2*, and *EIN4*; ethylene signaling genes, such as *EIN3*, *ERF1*, *ERF2*, and *CTR1*; and MAPK cascade genes, such as *MEKK1–MKK2–MPK4/6*, are modulated in cotton under both short and long salt treatment periods [6]. In contrast, *NTHK1* interacts with an ankyrin domain-containing protein *NEIP2* (*NTHK1*), an ethylene receptor-interacting protein, to improve salinity stress tolerance in tobacco [138]. These findings suggest the negative regulation of ethylene receptors in salinity stress tolerance and indicate ethylene as a positive mediator of salinity stress tolerance in plants. Moreover, many salt concentrations stimulate EBF1/2 deprivation and increase the accumulation of the EIN3 protein in an EIN2-independent manner [6]. In addition, salt stress increases *EIN3* transcriptional activity in an EIN2-dependent manner in rice [139]. ETR1 and ERS1 mediate ethylene and H_2_O_2_ signaling, highlighting the ethylene-mediated regulation of H_2_O_2_ concentrations during salinity stress in the guard cells of *Arabidopsis* leaves [140]. Further, salt stress up-regulates the genes associated with Ca^2+^ signaling and ABA biosynthesis in the leaves of *ETR2B*, demonstrating the ethylene receptor’s role in salt stress intercede by Ca^2+^ and the ABA signaling pathways in *Cucurbita pepo* [141]. Ethylene and salt stress in plants confirm that ethylene levels might positively or negatively affect plants’ responses to salt stress, suggesting that the fine-tuning of ethylene action might be necessary for salt stress tolerance in plants.

The induction of *ERF1* gene expression after salt and dehydration stress is enhanced by ethylene signaling [111]. *Arabidopsis* with a constitutive promoter, *35S:ERF1*, is drought-tolerant and exhibits gene expression and regulation under drought with a higher survival rate, leading to a low yield [3,142]. The study by Arraes et al. [142] stated that the ethylene gene *CTR* involved in signal transduction is downregulated under drought conditions. The ethylene response factor (*OsERF109*) negatively affects rice’s ethylene biosynthesis and drought tolerance [143]. Under drought stress, the *eto 1* mutant of rice has an insensitive *OsETOL1* protein with a high ethylene level, which helps the plant to survive [142]. Ethylene is involved in opening and closing the stomatal aperture in *Arabidopsis* mutant *eto1* [144]. Interestingly, ethylene response factors *EIN3* and *ETR1* (responsible for ethylene signaling) are not involved in the stomatal closing movement. When plants are exposed to drought, ethylene causes leaf abscission, reducing water loss [145]. Under drought, the production of ethylene increases and results in an initial increase and subsequent decrease in ACC, suggesting that drought induces the de novo synthesis of ACS, the rate-controlling enzyme of the ethylene biosynthesis pathway [142]. Under flooding, increased ethylene production results in leaf abscission and senescence, seed germination, growth of adventitious roots, epinasty stimulation, and inhibition of shoot growth, as well as stomatal closing and flowering [146]. Ni et al. [147] reported that ethylene released under waterlogging stress in *Helianthus annuus s*tems results in aerenchyma formation to facilitate gas exchange. This is mediated by ethylene, which induces ROS signaling and mediates aerenchyma formation through programmed cell death (PCD).

Transgenic tobacco plants with reduced ethylene biosynthesis under salinity stress show enhanced tolerance [148], and, upon exogenous supplementation of ethylene, rice shows hypersensitivity to salinity [108,149]. Similarly, increased ethylene production in rice under salt stress reduces plant growth, grain filling, and the development of spikelets [150], which are reversed by the application of 1-MCP (ethylene action inhibitor), in rice under salinity stress [151]. We cannot say that ethylene positively affects abiotic stress tolerance or negatively affects it unless we explore the whole mechanism that is affected under abiotic stress, including oxidative stress and antioxidative metabolism.

**Figure 4:**
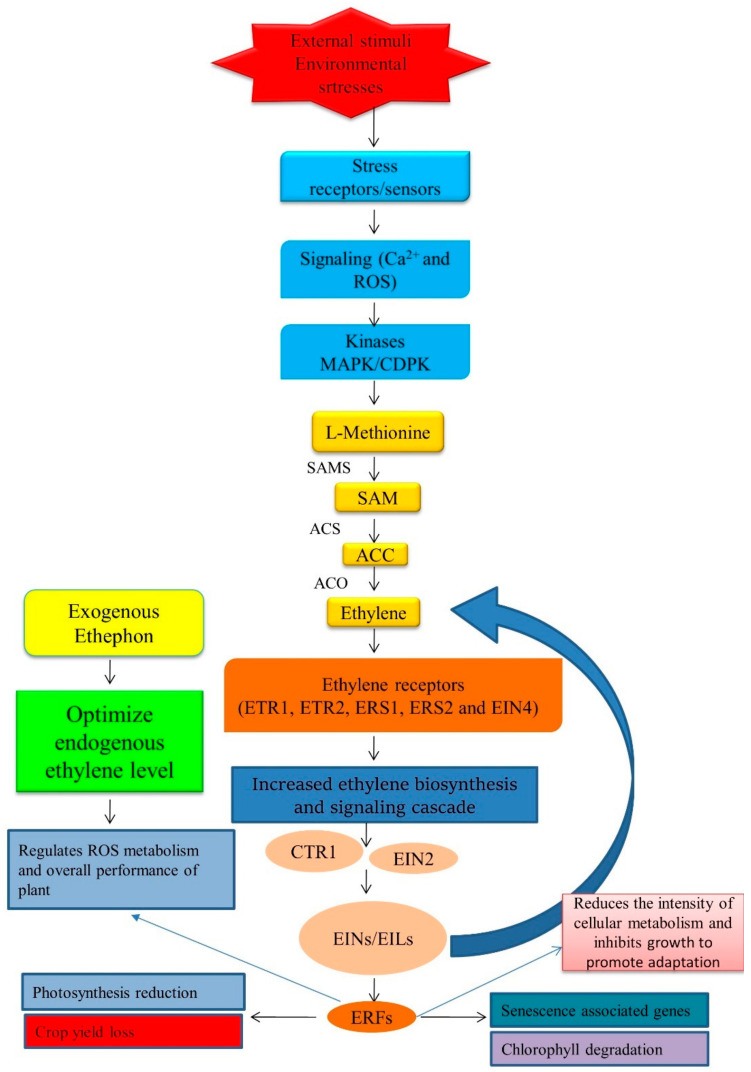
Regulation of ethylene biosynthesis and signaling for environmental stress acclimation. ACC, 1-aminocyclopropane-1-carboxylic acid; ACS, ACC synthase; CDPK, calcium-dependent protein kinase; Ca^2+^, calcium signaling; CTR1, CONSTITUTIVE TRIPLE RESPONSE1; SAM, S-adenosylmethionine; SAMS, SAM synthetase; ETR1, ETHYLENE RESPONSE1; ERS1, ETHYLENE RESPONSE SENSOR1; EIN4, ETHYLENE INSENSITIVE4; EIL, ETHYLENE INSENSITIVE3-LIKE 1; ERFs, ethylene-responsive factors; ROS, reactive oxygen species; MAPK, mitogen-activated protein kinase.

Ethylene accumulation plays a positive role in surviving flooding stress. In contrast to other stresses where gas diffusion is not impaired, gas exchange is severely restricted between the plant and its environment under flooding. Submergence promotes the biosynthesis of ethylene, which is entrapped in plant tissues, rapidly reaching response-saturating levels within 1–2 h [152,153]. This will initiate flood-adaptive responses. Ethylene moderates shoot growth towards the water’s surface, facilitating gas exchange with the atmosphere, developing adventitious roots, and maintaining the basic metabolism required to survive under submergence [101]. It has been suggested that utilizing the microbial enzyme ACC deaminase significantly decreases the ACC levels in plants, especially plants subjected to flooding stress, thereby decreasing the amount of stress ethylene and the subsequent damage to the plant by stress ethylene [103]. Accordingly, Huang et al. [154] stated that ethylene signaling bears importance in the survival of rice seedlings under submergence. It was revealed submergence activates ethylene signaling through the slight down-regulation of *CTR1* and up-regulation of *EIN3* genes via in silico analysis [155]. Moreover, ethylene ameliorates ROS during subsequent reoxygenation [101]. Interestingly, ethylene-induced adventitious root growth is mediated by the ROS activity of antioxidant enzymes in rice [124]. Further, several ERFs and genes aid rice plants under flooding conditions by enhancing the GA action essential for stem elongation [156]. Major ethylene signaling components (EIN2) and several AP2/ERF transcription factor gene family members have regulatory roles in plants under environmental stress [157]. Indeed, *OsEIL1* is involved in the ethylene signal transduction pathway and positively regulates the ethylene response in rice [158]. The ethylene receptor in rice, *OsETR2*, is highly expressed in the internodes and is also induced by ethylene [159]. It has been found that the overexpression of *OsETR2* reduces the ethylene concentration, and conversely, *OsETR2* knockout plants show enhanced ethylene sensitivity in rice [160]. Thus, ethylene plays a notable role in plant adaptation to environmental stress by regulating several processes that influence plant growth and survival, including biosynthesis and signaling pathways (Figure 4). Table 1 shows some selected studies on the response of plants to ethylene modulators under major environmental stresses.

Ethylene in excess restricts plant growth by reducing the effect of auxins on the epidermal layer, which senses the environment and subsequently drives the growth of the inner tissues [168]. Mainly, it restricts plant growth by inhibiting cell elongation in crosstalk with auxin. At very low concentrations, ethylene is biologically active, and its sensitivity depends on the species and affects the plant response [169]. Organ or plant growth are controlled by affecting processes, such as cell division, expansion, or differentiation in different tissues [170], and ethylene potentially affects these functions. Studies have found that, in *Arabidopsis*, ERF5 and ERF6 improve leaf growth under environmental stress [171]. However, this is concentration and species-dependent [172,173]. There is a contrasting effect of ethylene on slow-growing and fast-growing *Poa* species, where slow-growing species are more responsive to ethylene and show greater leaf elongation inhibition compared with fast-growing species. However, a low level of ethylene promotes leaf elongation in slow-growing species, while slight inhibition occurs in fast-growing species at the same concentration [173]. Ethylene reduces the leaf area, perhaps due to leaf epinasty, which reduces light capture and thus reduces CO_2_ assimilation. Ethylene could regulate the growth of the leaf by inducing ROS, and nitric oxide (NO) is also involved in leaf expansion [174]. Thus, ethylene can act as a positive or negative regulator of growth, depending upon the ethylene concentration, plant type, and organ under study.

## 5. Potential Mechanisms Underlying Ethylene-Mediated Environmental Stress Tolerance and Plant Responses

Studies have suggested a potential role of ethylene in the synthesis of secondary metabolite and osmolytes and antioxidant metabolism under optimal and stressful conditions [165,175]. Ethylene also interacts with several nutrients and phytohormones, and modulates antioxidant defense and plant metabolism [14,176]. Therefore, this section explores the mechanisms underlying ethylene-mediated environmental stress tolerance and plant responses.

### 5.1. Ethylene Interaction with Secondary Metabolites, Osmolytes, and Carbohydrates

Secondary metabolites are substances that are biosynthetically derived from primary metabolites and produced by plants for defense. These metabolites are involved in plant metabolic activity. Secondary metabolites are beneficial for plant tolerance under stressful environments [8,177]. A recent study by Ma et al. [17] stated that melatonin primarily regulates the pathways of plant hormone signal transduction and secondary metabolite biosynthesis via ethylene, and suggested that ethylene is involved in the induction of secondary metabolite synthesis in plants. Melatonin induces the expression of *VvMYB14*, which increases ethylene production by transcriptionally activating *VvACS1* and thereby affects the accumulation of secondary metabolites. Exogenous ethylene enhances Cd resistance by influencing terpenoid indole alkaloids’ biosynthesis in *Catharanthus roseus* seedlings [178]. Exogenous ethylene upregulates the transcriptional expression of metallothionein and increases Cd accumulation in leaves. Papon et al. [179] reported that the ethylene signaling pathways and elements of cytokinin improve the production of terpenoid indole alkaloid through metabolic engineering. Ethylene combined with methyl-jasmonate (Me-JA) on phenolic compounds’ metabolic profiling in *Pinus albicaulis* suggests that the ethylene–MeJA combination controls phenolic metabolism [180]. Moreover, the ethylene response factor *NtERF91* positively regulates alkaloid accumulation in tobacco [181]. These findings suggest that ethylene regulates the biosynthesis of secondary metabolites. The biosynthesis and accumulation of metabolites rapidly change during stress to overcome the adverse effects of environmental stresses and play a role in plant stress tolerance. A recent study of Yadav et al. [182] reported that drought stress triggers the synthesis of secondary metabolites and secondary metabolite-mediated antioxidant machinery to scavenge ROS. They conducted expression analysis of genes encoding transcription factor *ERFs*, and found that they play a crucial role in drought response.

Ethylene regulates environmental stress tolerance by influencing different osmolytes [183]. Studies on *ein2-5*, and *ein3-1* (ethylene insensitive) mutants also confirmed the involvement of ethylene in osmolyte biosynthesis. Ethylene increases the accumulation of compatible solutes and decreases oxidative stress to improve plant tolerance to water stress in *Arabidopsis* [166]. Osmolytes contribute to ROS detoxification induced by oxidative stress and increase the plant response to long-term stress. Amphoteric quaternary amines (glycine betaine) regulate water balance in a plant cell by stabilizing cellular structure and activity under drought stress [184]. Further, the enhanced accumulation of glycine betaine and reduced ethylene under salt stress increase the GSH content, lowering oxidative stress in rice [156]. Ethephon increases the photosynthetic-N-use efficiency, and proline and antioxidant metabolism to alleviate a decrease in photosynthesis under salinity stress in mustard [185]. The study of Nazar et al. [110] reported that drought stress-induced ethylene production is reduced, while proline accumulation and drought tolerance are improved with salicylic acid (SA). Further, proline plays a role in stress tolerance mechanisms as an ROS scavenger [185].

The exogenous application of ethephon improves carbohydrate metabolism under heat stress. Ethylene application reduces oxidative stress by enhancing enzymatic antioxidants and significantly increasing the sucrose and starch contents, and carbohydrate metabolism enzyme activities for heat tolerance in rice [15]. These soluble sugars work as a signal molecule to regulate different gene expressions involved in photosynthesis [184] and act directly as negative signals or modify the cell pathways to induce stress response signals and increase plant resistance to stress [186]. In another study, exogenous ethylene inhibited sprouting potato tubers by influencing their carbohydrate metabolism [187]. Endogenous ethylene regulates *Medicago sativa* embryo germination and influences the activity of α-amylase and the metabolism of soluble carbohydrates [188]. The study by Wang et al. [189] stated that ethylene significantly increases the fructose and glucose levels, but does not affect the sucrose and total soluble sugar contents in ripe jackfruit postharvest. Interestingly, fructose and glucose are hexoses, causing a massive alternation in sugars compared with cyclitol and scylloinositol. Glucose directly helps in the osmotic adjustment in different species, such as oak pine, but cyclitols primarily help to protect and stabilize the DNA structure under drought stress [190]. Ethylene increases glucose utilization by influencing the photosynthetic potential and sink strength, and reduces the glucose-mediated repression of photosynthesis in wheat under salt stress [165]. Moreover, the ethylene receptor *ETR2* delays flowering and increases starch accumulation in stems by downregulating the genes in rice [160]. Interestingly, starch is emerging as a key molecule in mediating responses to different environmental stresses. Plants generally remobilize starch to provide energy and carbon during photosynthesis. The released sugars and derived metabolites support plant growth and development by mitigating the negative effect of stresses in ripening jackfruit [189].

### 5.2. Ethylene in the Modulation of Enzymatic and Non-Enzymatic Antioxidants and Phytochelatins

The involvement of ethylene in the modulation of enzymatic and non-enzymatic antioxidants has been widely reported to control plant tolerance to various environmental stresses [7,16,165]. Environmental stresses cause excess ROS generation and produce oxidative stress conditions. The application of ethylene alleviates the adverse effects of salt stress on the photosynthesis and growth of plants by enhancing the activity of enzymatic antioxidants, such as superoxide dismutase (SOD), ascorbate peroxidase (APX), and glutathione reductase (GR) [176]. Ethylene also modulates H_2_O_2_-metabolizing enzyme activities, such as those of catalase (CAT) and APX, in plants exposed to salt stress [10]. Under Cd stress, ethylene is involved in selenium-induced defense responses through the antioxidant system [191]. Ethylene and S applications are responsible for lowering ethylene formation to the optimal range and synthesizing the maximal GSH production to protect the plants from Cd-induced oxidative stress [113]. It has been reported that the S-containing metabolite methionine is a precursor of ethylene and is a rate-limiting metabolite for ethylene production [113]. There is an interrelationship between S assimilation and ethylene signaling responsible for plant vigor under optimal and stressful environments [4]. Under salt stress, ethylene regulates a plant’s responses to excess S and increases the contents of cysteine (Cys) and GSH [7]. It has been suggested that ethylene works as a stimulant for producing pharmaceutically important non-enzymatic antioxidants in chamomile leaves [192]. Indeed, ethylene also regulates antioxidant metabolism under salt stress to increase the photosynthetic potential of mustard grown with optimum and low N levels [185].

Interestingly, another potential mechanism underlying ethylene-mediated environmental stress tolerance is the activation of the elements in the promoter region of the heavy metal-responsive genes and the metal chelator phytochelatins [193]. Phytochelatins are synthesized from GSH and play an important role in the heavy metal defense mechanism. It has been reported that ethylene influences phytochelatin synthesis at the molecular or gene expression level. The report by Keunen et al. [89] suggested that several toxic metals induce ethylene biosynthesis and signaling in various plant species, but it depends on the plant species and type and concentration of heavy metal treatment. It was also described that the induction of stress ethylene causes non-beneficial symptoms in plants, and the application of an ethylene inhibitor completely reduces the negative effect of heavy metals [194]. The absence of ethylene (due to an ethylene biosynthesis inhibitor) causes a decrease in the phytochelatin synthesis activity and the amount of –SH groups in plants [195]. However, only a few reports are available on the direct relationship between phytochelatin and ethylene under heavy metal stress; therefore, it is more likely that its possible role in ethylene signaling under different environmental stresses awaits further scrutiny.

In *Arabidopsis*, *ETO*1 increases ROS formation and maintains Na^+^/K^+^ homeostasis. Under different stress conditions and during various growth stages, ethylene-regulated downstream signaling generally relies upon ROS [3], and increased ROS signals for an increase in antioxidative enzymes. In *Arabidopsis* exposed to ozone stress, GSH synthesis is enhanced, which is regulated by ethylene [18]. Ethylene is thought to be involved in the Se-induced antioxidant system that improves growth under Cd stress [191]. Ethylene triggers the antioxidative defense system in plants, which reduces oxidative stress and maintains growth and photosynthetic efficiency [183]. ACC, which acts as an ethylene precursor, enhances the activities of the antioxidant enzymes APX, CAT, SOD, and POX and reduces lipid peroxidation in plants [88]. Takács et al. [107] reported that ethylene regulates ROS metabolism by modulating antioxidant enzymes. Ethylene-induced ERFs are known to play a role in redox regulation and are themselves induced by stress [108].

### 5.3. Ethylene and Nutrient Responses

The availability of mineral nutrients plays a vital role in plants’ optimal growth and development. Several reports demonstrated an association between ethylene and nutrient metabolism under environmental stresses [4,8,90]. Mineral nutrients influence ethylene biosynthesis by supporting a close interaction between ethylene stimuli and nutrient homeostasis, such as ethylene and S [10], ethylene and K [196], ethylene and calcium (Ca) [197], ethylene and N plus S [14], etc. Ethylene interacts with nutrient uptake, such as that of S and N, minimizes oxidative stress, and controls plant responses under optimal and stressful conditions [10,13,185]. A recent study by Yu et al. [198] stated that Ca signaling plays a vital role in the adventitious root formation under salt stress by regulating endogenous ethylene synthesis and the signal transduction pathway in cucumber. Ethylene has also been implicated in regulating physiological and morphological plant responses to nutrient deficiencies. The deficiency of mineral nutrients appears to interact with ethylene production and sensitivity. Ethylene is essential in regulating phosphorous deficiency and induces developmental and physiological changes [199]. Ethylene also mediates root responses to boron deficiency [200]. Similarly, in *Arabidopsis*, ethylene is related to N deficiency [201], inducing the ethylene production signaling pathway. Ethylene regulation has also been extended in response to other deficiencies, such as K, S, and many more [4,202,203]. Moreover, ethylene signaling is responsible for root morphology and whole plant tolerance changes to low-K conditions [196].

### 5.4. Ethylene Crosstalk with Phytohormones

Several plant hormones, such as auxin, cytokinin, ABA, GA, SA, etc., can influence ethylene production [11,16,22,159,166,204]. In particular, the hormones auxin and cytokinin impact ethylene evolution more than the other hormones. The increment in auxin stimulates ethylene evolution by de novo ACS synthesis. Likewise, cytokinins in a high amount increase ethylene production. Ethylene production is noteworthy with auxins and cytokinins compared with the hormone alone. It has been reported that the developmental plasticity of the plant root is regulated by the auxin–ethylene interaction under high salinity and water stress, and the stress-induced remodeling of root architecture confers stress tolerance in plants [205]. It has also been reported that ethylene signaling acts synergistically with auxin to mediate aluminum (Al) stress-induced root growth inhibition in *Arabidopsis* [206]. Notwithstanding, it is unclear whether endogenous ABA limits ethylene synthesis precursor ACC or the change of ACC to ethylene. Ethylene prevents polar auxin transport, although the hormone indole acetic acid (IAA) stimulates ethylene synthesis. Accordingly, ethylene might bring down the measure of operative IAA, which might diminish the step of ethylene synthesis. The significant collaborative impact of IAA and ethylene appears to be responsible for reducing the tissue response to ethylene. Mathieu et al. [207] showed that ABA and JA are more noteworthy in this process; however, the synthesis of ACC is lower in chicory plants. Ethylene emanation remained continually high during the 5 h examination, yet ABA, SA, and JA diminished in *Melissa officinalis* leaves [208]. The concentrations of ABA, ACC, and JA, were essentially greater in leaves; in contrast, the IAA and cytokinins diminished in plants pre-accustomed to high temperatures [209]. Cytokinin and ABA inhibited primary root growth by regulating ACS at the post-transcriptional level with increased ethylene synthesis. It has been reported that the increased ethylene due to Al stress increases the expression of cytokinin biosynthetic genes encoding IPTs, leading to root growth inhibition [210]. Interestingly, the overexpression of cytokinin repressor genes, *ARRs*, serves as a mediator of the crosstalk between ethylene and cytokinin in the cold stress response and confers freezing tolerance in *Arabidopsis* [211]. It has also been suggested that the knockouts of ethylene biosynthesis and signaling genes in *Arabidopsis* result in altered plant sensitivity to ABA and, accordingly, affect the ABA-dependent abiotic stress tolerance in several developmental processes. For example, the *Arabidopsis* loss-of-function mutant (*acs7*) with reduced endogenous ethylene levels is hypersensitive to ABA, accumulates higher levels of endogenous ABA, and displays enhanced salt tolerance during seed germination and seedling growth [212]. Moreover, it has also been reported that both ethylene and ABA act antagonistically in controlling plant growth and development and under abiotic stress conditions [213]. Ethylene application with S coordinately modulates the accumulation of ABA for salinity stress tolerance in mustard plants [10]. Higher brassinosteroids promote ethylene biosynthesis via enhancing ACS, but are repressed by ABA [22,204]. It has also been reported that both ethylene and brassinosteroids are involved in plant responses to environmental stresses [114,214]. Brassinosteroids induce ethylene biosynthesis and ROS generation, which subsequently enhance the AOX capacity, leading to increased salt and drought stress tolerance in *Cucumis sativus* [215]. Brassinosteroids ameliorate the inhibitory effect of salt by regulating ethylene production via the recovery of the NaCl-induced suppression of ACO activity in ethylene production [216]. Moreover, brassinosteroids affect ethylene biosynthesis and signaling by increasing ACS and stabilizing EILs in *Solanum lycopersicum* under salinity stress [217]. Major ethylene and JA signaling hubs, such as EIN2, EIN3, and AP2/ERF, and the JAZ proteins CTR1 and MYC2, have complex regulatory functions during the adaptation of plants to abiotic stress [157]. It has been shown that EIN3/EIL1 positively regulates JA-dependent root hair development and thus enhances drought tolerance in *Arabidopsis* [166]. Ethylene and NO interact to regulate magnesium deficiency-induced root hair development in *Arabidopsis* [218]. Under changing environments or abiotic stress, the ethylene and NO interaction mechanisms contribute to the stress tolerance of crops with increased yield [219]. It has been shown that SNP (NO donor) induces *MfSAMS1* expression, resulting in an elevated SAM level, polyamine concentration, and polyamine oxidation under cold stress in *Medicago sativa*. Similarly, altered ethylene emissions have been observed parallel with the enhanced tolerance to cold stress [220]. This report indicated that SAM synthetase plays an important role in plant tolerance upon cold stress by up-regulating polyamine oxidation and improving H_2_O_2_-induced antioxidant protection. Moreover, transient NO and ROS production and down-regulated *ETR1* expression have been observed after treatment with the non-protein amino acid β-aminobutyric acid, improving the drought tolerance of *Solanum tuberosum* [219]. Further, the interaction between ethylene and GA alleviates Cd stress in mustard by inducing S-assimilation [16]. Moreover, Masood et al. [221] also stated that ethylene and GA interplay in the regulation of photosynthetic capacity inhibition. Both these hormones, ethylene and GA, influence each other’s actions; GA is known to increase ethylene synthesis. On the other hand, its signaling is affected by ethylene; therefore, this interaction affects stress tolerance in plants [221]. A recent report showed that SA-supplemented plants exhibit reduced stress ethylene generation under heat exposure by decreasing the ACC and ACS activities to an appropriate range [222]. Interestingly, ethylene intervenes in SA’s effect in the presence of sulfate to induce S-assimilation, upregulates the antioxidant system, and imparts salt tolerance in plants [166].

## 6. Summarizing Ethylene Formation under Stress and the Mechanism of Action

Ethylene production increases in plants under stress. The same mechanism that produces ethylene under normal conditions produces excess ethylene under stress [223]. However, the process varies regarding the activation of the ACS by stress. Stearn and Glick [223] reported that, in the presence of stress, there is a small peak in the ethylene content within a few hours of exposure, which utilizes the existing ACC present in plants, but then, after a few days of stress, the level of ethylene sharply rises. The enhancement of ACC formation and ACS activity, and the rise in the ethylene level are the key reasons. Thus, as we know that ACS formation is activated by MAPK [122], which itself is influenced by stress, the ethylene level increases under stress. Summarizing our knowledge of ethylene signaling, it can be said that the ethylene response occurs by binding the ethylene to receptors and thus silencing them, facilitating ethylene responses. Under stress, this ethylene response aids in plants’ adaptation until the burst of ethylene occurs, which causes senescence and plant death. In leaves of plants exposed to environmental stress, ethylene appears to have a negative effect on the cell cycle [9]. It plays a dual role under stress when it regulates a defense response in fully grown leaves, but a growth response in young leaves [9]. A high level of ethylene in leaves causes the rapid inhibition of cell division and cell expansion, which could occur either through DELLA-mediated mechanisms or through the expression of *EXPANSIN* genes in the cell cycle. Reduction in growth under stress is a favorable response because it reduces the wastage of energy when plants are coping with a change in conditions that disturbs homeostasis. Thus, although excess ethylene is harmful to plants, a lower ethylene level initiates growth responses, as in the case of young leaves mentioned by Dubois et al. [9]. The application of ethephon induces the release of ethylene and controls the endogenous ethylene level and function [224]. The inhibition of excess ethylene is favorable, as has been reported under various stress conditions. The application of SA suppresses ethylene formation under drought stress, which results in increased photosynthesis and growth [225]. Ethephon treatment results in an increase in the GSH content and antioxidative metabolism, which lead to stress removal and subsequently increased photosynthesis and growth [15,165]. The initial rise in ethylene during stress is a signaling response to initiate defense in plants, which is concurrent with the rising ROS level and a subsequent increase in antioxidants. It may be said that ethylene can promote growth if the level of ethylene is kept at the initial peak level, which is generally achieved through the application of an ethylene biosynthesis inhibitor that reduces the ethylene level, or through the use of chemicals that either inhibit the ethylene level or alleviate antioxidative mechanism. Ethylene in itself is a beneficial signaling molecule for plant growth, provided that it is kept at an optimum concentration. In several studies, ethephon application has been shown to inhibit the release of ethylene [15,121]. Upon ethephon addition, the ethylene released binds to receptors and initiates signaling. It directs GSH synthesis, and the pool of methionine that was being utilized for SAM synthesis to form ethylene is now diverted to GSH, leading to a low ethylene level even under stress [122]. GSH pre-treatment reduces ROS and inhibits the activation of MAPK3 and MAPK6, which are reported to be involved in the regulation of ethylene biosynthesis and signaling in Cd-stressed *Arabidopsis* [226,227,228].

The response of ethylene under different abiotic stresses varies. It may, on the one hand, increase the antioxidative enzymes that scavenge ROS and reduce oxidative stress in plants to protect proteins, nucleic acid, and membranes from damage. On the other hand, it may regulate osmolytes, nutrients, and other phytohormones for stress tolerance. Depending upon the type of stress, the response of ethylene also varies. It was found that ethylene regulates ROS signaling, which enhances antioxidants, but under conditions of drought and salt stress, ethylene rather plays a defensive role that leads to plants’ adjustment under stress. It may enhance senescence, reduce stomatal opening, or increase the growth of adventitious roots that aid in water absorption under limited water availability. Stomatal closure stops water loss, while senescence may lead to the redistribution of nutrients in the sink organ. Ethylene has been shown to take part in enhancing sink activity [229]. Thus, the role of ethylene under stress could be promoting, reducing, or adjusting against stress; however, unequivocally, it always plays a role in abiotic stress; therefore, it becomes crucial to study the different stress-response proteins and their influence on ethylene under different abiotic stresses. The impact of ethylene on root architecture under different stress and whether it is acting in promoting or inhibiting growth should receive focus. If it increases growth, then what is the mechanism involved, and if it inhibits growth, then what are the processes involved should be explained. This will completely clarify the role of ethylene under different stresses. The interaction of ethylene with different plant growth regulators should also be explored, as we know that ethylene affects various growth regulators that are involved in stress tolerance.

## 7. Conclusions

The regulation of ethylene biosynthesis and the signaling transduction pathway by the influence of environmental stress and the influence of ethylene on stress responses has been discussed in this review. The optimum level of ethylene biosynthesis range and signaling pathway regulate various metabolic processes and modulate the antioxidant defense in plants, which varies from plant to plant. Ethylene interacts with secondary metabolites, osmolytes, and carbohydrates, and induces potential mechanisms in plants for stress tolerance. The interaction of ethylene with nutrients and phytohormones helps to promote plant growth by inducing plant metabolism.

In summary, this review provides insights into the role of ethylene signaling in regulating plant processes and developing sustainable crop production under optimal and environmental stress conditions. The collaborative knowledge and new perspectives of ethylene signaling will play a vital role in regulating plant growth and development, as well as stress tolerance to promote survival and acclimatize to varying environments. Detailed studies on ethylene biosynthesis and signaling could contribute to the applications of biotechnological strategies for developing improved and new variants of crops in response to different environmental stresses. Ethylene application to various types of agricultural interest may be expanded in the coming years for agribusiness purposes. The future concern is to focus on identifying ethylene’s antagonistic and synergistic role with other signaling cues and uncover more factual data on ethylene’s interplay with other hormones and environmental factors.

## Figures and Tables

**Table 1:** Selected studies on the response of plants to ethylene modulators under different environmental stresses.

Plant Name	Ethylene Concentration/Modulators	Stress Type	Parameter Studied	Response	References
Heat stress					
Oryza sativa	10 μM ACC	Heat stress	Heat shock factors, such as HSFA1a and HSFA2a, c, d, e, and f, and ethylene signaling genes	Increased tolerance	[112]
*Oryza sativa*	1.6 mM ethephon	Heat stress	ACS, ACO gene expression, and *psbA* and *psbB* genes	Decreased tolerance compared with increased stress plant	[15]
Heavy metal stress					
*Brassica juncea*	200 µL L^−1^ ethephon	1.2 mM Cr and 50 µM Cd	ATP-sulfurylase, serine acetyl transferase, and GSH	Increased tolerance	[5,90]
*Brassica juncea*	200 µL L^−1^ ethephon	50 µM Cd	Net photosynthesis Ethylene content and GSH content	Increased tolerance	[125]
*Brassica juncea*	200 µL L^−1^ ethephon	50 µM Cd	Ethylene, glucose, ACS, and increased PSII activity (except NPQ)	Increased tolerance	[90]
*Arabidopsis thaliana*	20 µM ACC	400 µM Cr	H_2_O_2_ accumulation (Staining)	Increased tolerance	[161]
*Arabidopsis thaliana*	20 µM Ag and Co	400 µM Cr	H_2_O_2_ accumulation (Staining)	Decreased tolerance	[161]
*Arabidopsis thaliana*	0.5 µM ACC	75 µM CdCl_2_	Superoxide accumulation	Decreased tolerance	[162]
*Brassica juncea*	200 µL L^−1^ ethephon	200 mg kg^−1^ Zn	ACS activity and ethylene	Decreased tolerance compared with stress plants	[121]
Salt stress					
*Arabidopsis thaliana*	100 µM AOA and 100 µM ACC	100 mM NaCl	Antioxidant metabolism	No significant effect	[163]
*Amaranthus caudatus*	0.01, 0.1, and 0.3 mM ethephon	25, 75, and 125 mM NaCl	Seed germination	Increased tolerance	[164]
*Brassica juncea*	200 µL L^−1^ ethephon	100 mM NaCl	Reduced oxidative markers and increased antioxidants	Decreased tolerance Increased tolerance	[10]
*Triticum aestivum*	200 µL L^−1^ ethephon	100 mM NaCl	*psbA* and *psbB* expression	Increased tolerance	[165]
Drought, flooding, and cold stress					
*Triticum aestivum*	0.1 µM ACC	Mild drought	Relative growth rate	Increased tolerance	[84]
*Arabidopsis thaliana*	*Col-0*; *ein2-5ein3-1*	Drought stress	Proline, P5CS1 mRNA, and soluble sugar higher in Col-0Oxidative stress higher in *ein2-5ein3-1*	Increased tolerance	[166]
*Oryza sativa*	10 μM ACC	Flooding	Chlorophyll content	Increased tolerance	[154]
*Vitis amurensis*, *Vitis vinifera*	100 μM AVG	Cold	ACC and ACO contents, and ethylene production	Decreased tolerance	[167]

## Data Availability

Not applicable.
